# Complete response to pembrolizumab in recurrent nested variant of urothelial carcinoma

**DOI:** 10.1002/iju5.12334

**Published:** 2021-06-24

**Authors:** Kyotaro Fukuta, Hirofumi Izaki, Keito Shiozaki, Ryoichi Nakanishi, Tohru Inai, Hideyuki Kataoka, Eiji Kudo, Kazuya Kanda

**Affiliations:** ^1^ Department of Urology Tokushima Prefectural Central Hospital Tokushima Japan; ^2^ Department of General Medicine Tokushima Prefectural Central Hospital Tokushima Japan; ^3^ Department of Pathology Tokushima Prefectural Central Hospital Tokushima Japan

**Keywords:** bladder cancer, immunohistochemical staining, nested variant, pembrolizumab, urothelial carcinoma

## Abstract

**Introduction:**

The nested variant of urothelial carcinoma is rare and shows poor prognosis. We report a case of complete response to pembrolizumab in recurrent nested variant.

**Case presentation:**

A 50‐year‐old man visited another hospital with hematuria and weight loss. Clinical stage T4aN0M0 bladder cancer and acute renal failure were diagnosed. He was referred to our hospital and underwent radical cystectomy. Histological examination showed pathological stage T4aN2 nested variant of urothelial carcinoma. He received 3 cycles of gemcitabine and carboplatin adjuvant chemotherapy. However, para‐aortic lymph node metastasis appeared 7 months after cystectomy. He received pembrolizumab as systemic chemotherapy. After 10 cycles, the lesion remained undetectable and we evaluated the response as complete. He has received 18 cycles in total and no recurrences or metastases have been observed.

**Conclusion:**

Pembrolizumab may offer effective treatment for nested variant of urothelial carcinoma.

**Abbreviations**
CD8: cluster of differentiation 8
CD8^+^TILs: cluster of differentiation‐8‐positive tumor‐infiltrating lymphocytes
CT: computed tomography
eGFR: estimated glomerular filtration rate
FDG PET/CT: fluoro‐2‐deoxyglucose positron emission tomography/computed tomography
ICI: immune checkpoint inhibitor
MRI: magnetic resonance imaging
NVUC: nested variant of urothelial carcinoma
PD‐1: programmed death‐1
PD‐L1: programmed cell death‐ligand 1
RECIST: response evaluation criteria in solid tumors
TURBT: transurethral resection of bladder tumor
UC: urothelial carcinoma

**Keynote message**

The nested variant of urothelial carcinoma (NVUC) is an important and rare subtype with poor prognosis. This report describes a complete response to pembrolizumab in recurrent NVUC. Pembrolizumab may be effective for NVUC and immunohistochemical staining for cluster of differentiation‐8‐positive tumor‐infiltrating lymphocytes (CD8^+^TILs) may help predict response to pembrolizumab.

## Introduction

The NVUC, a subtype of UC, is rare. It makes up approximately 0.3% of all invasive UC and shows an aggressive clinical course and poor prognosis.^1^, ^2^ However, the treatment strategy of NVUC has not been determined. Pembrolizumab, a PD‐1 inhibitor, is an ICI approved in Japan for treating platinum‐refractory UC, and provides a better overall survival benefit compared to chemotherapy.^3^ However, no previous reports appear to have described the efficacy of pembrolizumab for NVUC. This case with complete response of recurrent NVUC to pembrolizumab is therefore presented.

## Case presentation

**Fig. 1:**
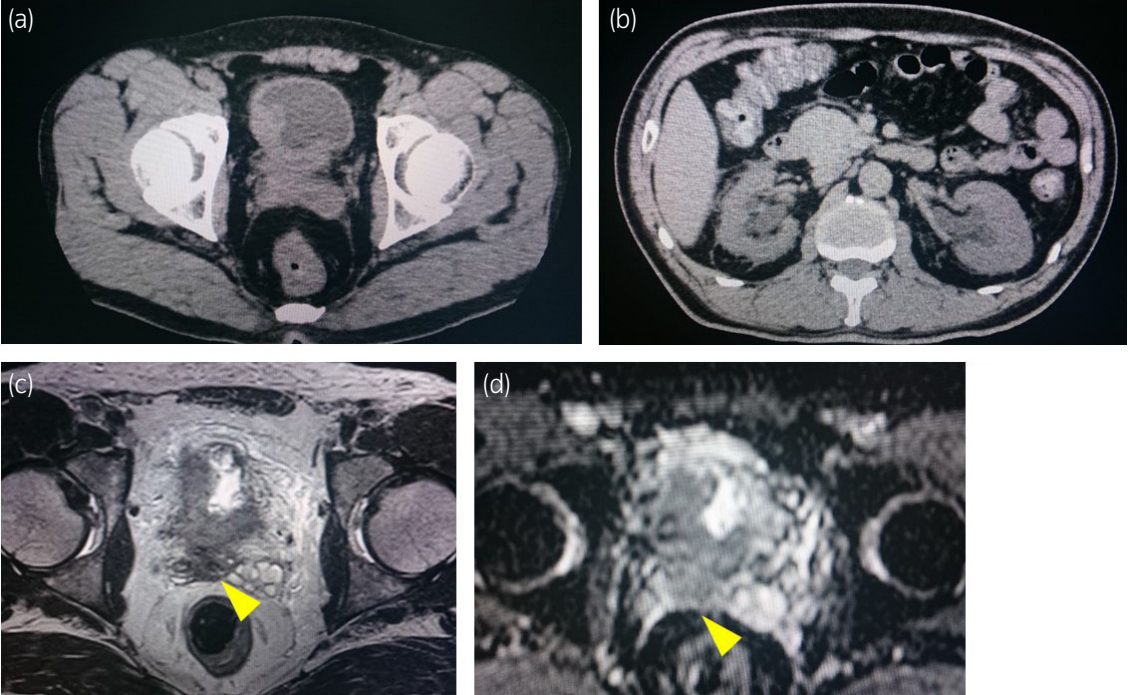
Results of preoperative abdominal CT (a, b) and pelvic contrast‐enhanced MRI (c, d). (a, b) Irregular thickening of the bladder wall (a) and bilateral hydronephrosis (b) are seen because bladder tumor involves bilateral ureteral orifices. (c, d) T2‐weighted imaging (c) and apparent diffusion coefficient mapping (d) show a large, irregular mass with significant extravesical invasion around the right wall and bladder trigone. Bladder tumor involves bilateral ureteral orifices and has invaded into the prostate and right seminal vesicle (yellow arrows). Clinical stage T4a bladder cancer was diagnosed.

**Fig. 2:**
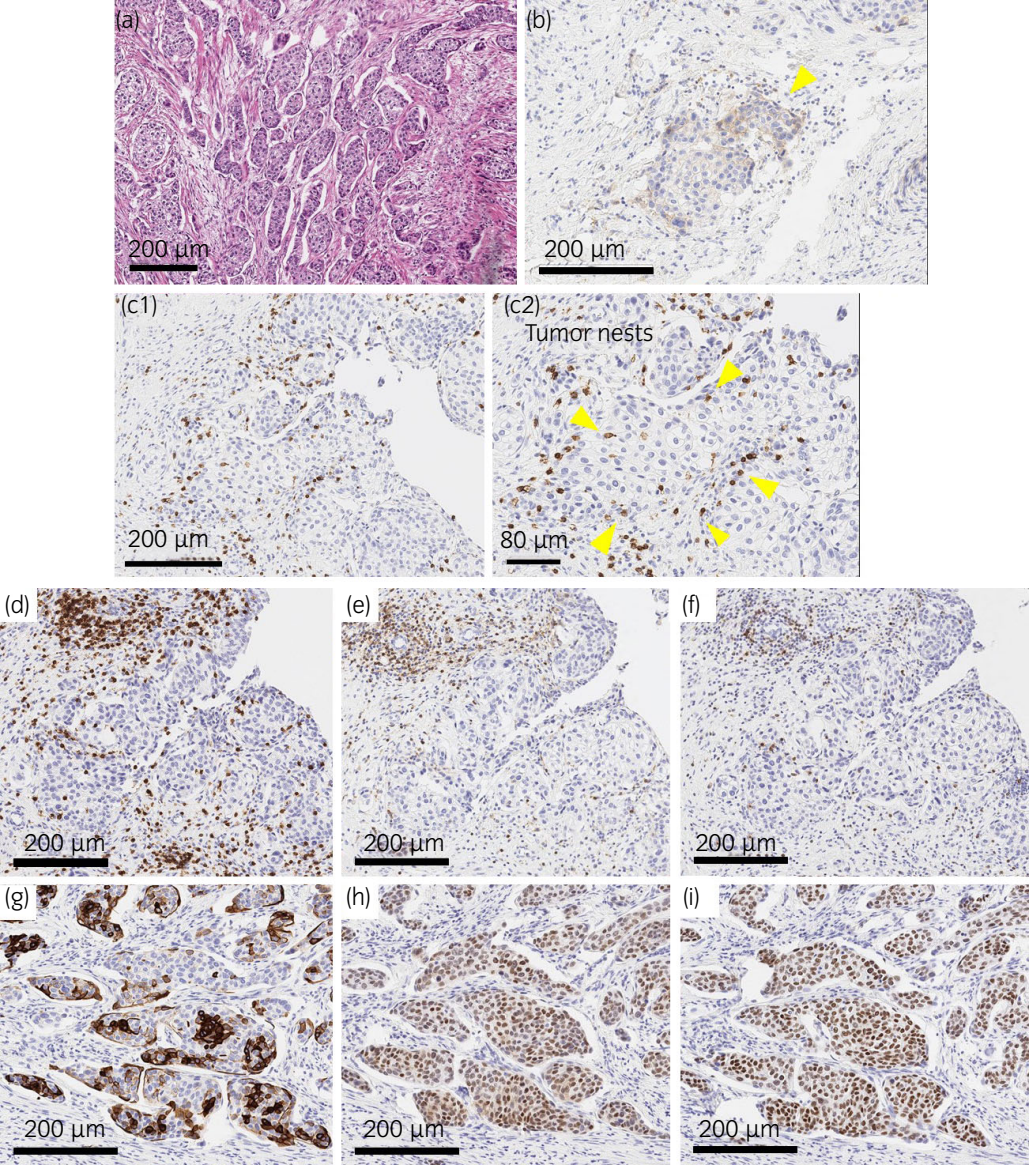
Pathological findings. (a) Hematoxylin and eosin staining of the bladder tumor. Most of the tumor cells formed small to medium‐sized nests that invaded the entire layer of the bladder. It was diagnosed as NVUC. The proportion of nested variant was more than 90%. (b) PD‐L1 immunohistochemical staining (anti‐PD‐L1 antibody (clone 73‐10), Leica Microsystems Inc., U.S.). PD‐L1 expression was approximately 1% (yellow arrows). (c‐f) Serial sections immunostained with CD8 (c1, c2), CD3 (d), CD4 (e), and PD‐1 (f). Most of the lymphocytes infiltrating in the tumor nests were CD8 positive (c1). CD8‐positive lymphocytes showed in the tumor nests at higher magnification (c2). Some of them seemed to be positive for PD‐1 also. (g‐i) Serial sections immunostained with Cytokeratin 5/6 (g), GATA3 (h), and p63 (i). Cytokeratin 5/6 was positive in a part of tumor cells in every tumor nests, whereas both GATA3 and p63 showed diffuse nuclear staining. These staining patterns were consistent with NVUC.

**Fig. 3:**
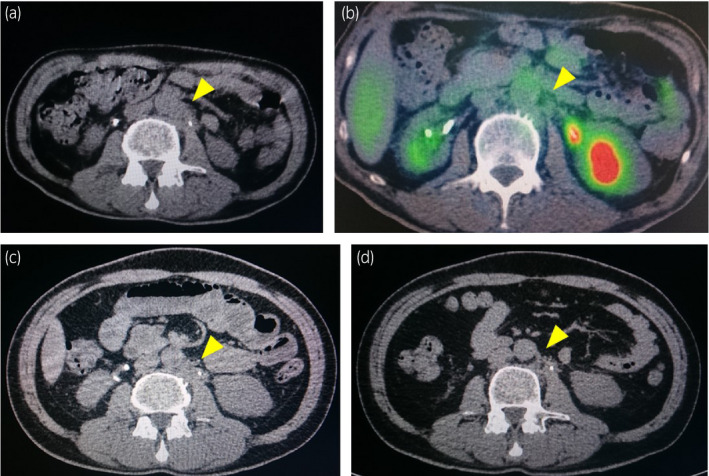
Findings from postoperative abdominal CT and FDG PET/CT. (a, b) Abdominal CT and FDG PET/CT show para‐aortic lymph node metastasis (yellow arrow) appearing 7 months after cystectomy. Increased accumulation (SUVmax 2.4, yellow arrow) is seen in the lesion on FDG PET/CT. (c) After 5 cycles of pembrolizumab therapy, the metastatic lesion (yellow arrow) had decreased in size. (d) After 10 cycles, the lesion remained undetectable on CT (yellow arrow). We evaluated the complete response by RECIST version 1.1.

**Fig. 4:**
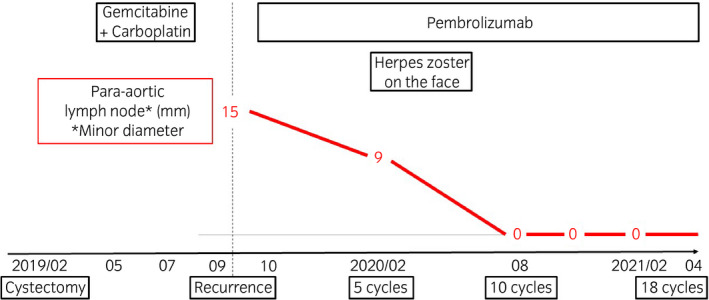
Clinical treatment course. He continues to receive pembrolizumab therapy even now.

A 50‐year‐old man with a history of heavy smoking visited another hospital due to gross hematuria and weight loss of about 10 kg for 3 months in January 2019. CT showed bilateral hydronephrosis and irregular thickening of the bladder wall (Fig. 1a,b). MRI showed a large irregular mass with significant extravesical invasion around the right wall, bladder trigone, and invasion of the prostate and right seminal vesicle (Fig. 1c,d). Laboratory data showed acute renal failure (creatinine, 7.01 mg/dL; eGFR, 7.51 mL/min/1.73 m^2^). Clinical stage T4aN0M0 bladder cancer and acute postrenal failure due to the mass were diagnosed. TURBT for diagnosis and left percutaneous nephrostomy were performed. Histopathology findings showed invasive UC, high‐grade, T2. Therefore, the patient was referred to our hospital for cystectomy. Renal failure did not improve sufficiently despite nephrostomy, so we performed robotic radical cystectomy and ileal conduit without neoadjuvant chemotherapy in February 2019. Histological examination showed high‐grade NVUC, T4a (Fig. 2a), and lymph nodes metastases (pathological stage N2). The nested variant represented more than 90% of the tumor. Since renal failure gradually improved after cystectomy (creatinine, 1.39 mg/dL; eGFR, 44.03 mL/min/1.73 m^2^), he received adjuvant chemotherapy with gemcitabine (1000 mg/m^2^) and carboplatin (area under the concentration–time curve, 4) for 3 cycles. However, para‐aortic lymph node metastasis appeared 7 months after cystectomy on CT and FDG PET/CT (Fig. 3a). He received pembrolizumab (200 mg) as systemic chemotherapy. After 5 cycles of pembrolizumab therapy, the metastatic lesion had decreased in size (Fig. 3b). After 10 cycles, the lesion remained undetectable (Fig. 3c). We assessed complete response based on the RECIST version 1.1. During pembrolizumab therapy, he developed herpes zoster on the face (Common Terminology Criteria for Adverse Events version 4.0, grade 2). He received 18 cycles of pembrolizumab therapy and continues the treatment. No recurrences or metastases have been observed (Fig. 4).

## Discussion

NVUC, a rare variant of UC, shows an aggressive clinical course.^1^, ^2^ It has been associated with muscle invasion on TURBT, extravesical invasion on radical cystectomy, and metastatic disease compared with pure UC.^2^, ^4^ Holmäng et al. reported that 7 of 10 patients who were treated with locoregional therapy died of disease or complications of therapy, 4–40 months after diagnosis.^1^ For locally advanced NVUC, radical cystectomy is the best choice, but the treatment strategy has yet to be established because of the rarity of this entity and the lack of randomized studies. Response to neoadjuvant chemotherapy was poor, at only 13%.^2^ Adjuvant chemotherapy and radiotherapy have not been shown to be significantly beneficial in reported cases.^1^, ^5^ The present case also represented locally advanced bladder cancer at initial diagnosis. The reason histological examination of the specimen from the initial TURBT did not diagnose NVUC was that they are not enough of the specimen for detecting muscle‐invasive UC. Early diagnosis is important, but to diagnose NVUC is difficult from specific findings on urine cytology and cystoscopy remain lacking.^6^

The PD‐1 inhibitor pembrolizumab as second‐line treatment for advanced/metastatic UC provides benefit in terms of overall survival and safety compared to chemotherapy according to the overall results of the KEYNOTE‐045 study.^3^ According to that study, pembrolizumab was associated with a benefit over chemotherapy in all the subgroups examined.^3^ The present case was in the following subgroups: current smoker; variant histology; and only lymph node metastasis. Pembrolizumab therapy may be effective and safe for patients not fit for cisplatin.

The duration of pembrolizumab therapy after complete response is uncertain. Y Fradet, et al. reported that median duration of response to pembrolizumab was not reached (range 1.6+ to 30.0+ months) on 57 patients who had evaluated complete or partial response.^7^ Therefore, we consider to continue pembrolizumab even he has remained in complete response until duration of pembrolizumab therapy is clear.

According to previous reports, the presence of CD8^+^TILs has been associated with favorable prognosis in colorectal cancer and urothelial cancer^8^, ^9^, ^10^, ^11^. Furthermore, another report indicated that expression of PD‐L1 and CD8^+^TILs is a predictor for response of some solid tumors to PD‐1 blockade therapy.^12^ We also performed PD‐L1, CD8, CD3, CD4, PD‐1, Cytokeratin 5/6, GATA3, and p63 immunohistochemical staining of specimens from cystectomy (Fig. 2b–i). All of the expression was positive. Especially we found significant numbers of CD8‐positive lymphocytes in the tumor nests, otherwise PD‐L1 expression of the tumor cells was low (tumor proportion score nearly 1%). In the KEYNOTE‐045 trial, pembrolizumab for advanced UC resulted in significantly improved overall survival regardless of tumor PD‐L1 expression status.^3^ Fumet et al. reported that CD8 expression was associated with good prognosis in PD‐1 blockade therapy for non‐small cell lung cancer.^13^ CD8 is an important marker of cytotoxic T cells, with effective functioning of CD8‐positive T lymphocytes as the main antitumor effector. In the present case, the high density of CD8^+^TILs might have contributed to response to pembrolizumab.

No previous reports appear to have described the efficacy of pembrolizumab for NVUC. To the best of our knowledge, this may be the first description of complete response to pembrolizumab in recurrent NVUC. Confirming the presence of CD8^+^TILs may predict the efficacy of pembrolizumab therapy.

## Conclusion

We encountered a case of recurrent NVUC with complete response to pembrolizumab. A high density of CD8^+^TILs may help predict response to pembrolizumab therapy.

## Conflict of interest

The authors declare no conflict of interest.

## Approval of the research protocol by an institutional reviewer board

Not applicable.

## Informed consent

Obtained from the patient.

## Registry and the registration no. of the study/trial

Not applicable.
